# Miscellanies

**Published:** 1869-10

**Authors:** H. Scott


### MISCELLANIES.

BY H. SCOTT.

I could not think of a heading that would appropriately cover the rambling thoughts I proposed to myself to write down, and which I hope to make suggestive of better conduct in some things pertaining to Dental practice, and I have, therefore, chosen the only one that is supposed to embrace everything. There are many matters I wish to speak of, and as I wish to be untrammeled, I shall let my thoughts take an entirely miscellaneous range. I don't intend to intentionally tread on any bodies toes, but if my strictures should fit any one, I have only to say that I hope such will be as much obliged to me as I would be to them if they were to tell me of my faults and short-comings.

I am not unaware that it is easier to give precepts than to execute them. I know that it is easy to run on over paper, penning plausible things, or verbally to magnify and overstate, while I am equally conscious that in either case it is practicable to confine one's self to facts and actual experience. Too much, perhaps, has been written that has done no body good; while on the other hand, too little of actual experience has found place in our journals. We don't want fancy or speculative theory, we want facts. Our books (some of them) abound with impracticable suggestions; suggestions that cause many to blunder in trying to reduce them to practice. And then, every one that thinks worth while to write, or speak his experience, has ways of filling teeth, and doing other things, peculiarly his own, just as if there were no one best method.

I know it may be said that some men can make good fillings by one method, and that others can make as good by another method; but all this looks to me like empiricism. One physician cures fever by alopathy, another by hydropathy, and still another by homeopathy. This seems very strange. It is incomprehensible, but we must let it pass. For myself I can conceive that there can be but one *methodis medendi* in specific disease, and but one best way of performing Dental operations, as there can be but one law governing the action of acids on alkalies. The only question is, are we, any of us, masters of our specialties? That's the inquiry. If we are not, we are to the extent that we fall short, groping in the dark. The writer is entitled to no exemption under his strictures, and only pleads a very long and careful observation as an apology for writing at all.

Let us have facts carefully and correctly stated so that any one can understand exactly what has been done, and what is meant. Some things in our literature are obscured by ambiguity; some are carelessly stated, while others are so delivered as to utterly bewilder the student. Thus, one writer said he had seen "whole sets of teeth hopelessly decayed, and judged to be unfit for filling, restored to health and made serviceable for the purposes of mastication by the use of the file alone." Now, is there a practical Dentist on earth who can tell what this man meant to say?

Another says he "continues the use of the file 'till the softened bone is all removed, as a preventative of decay." How would a young practitioner proceed under such a suggestion as this? And a "preceptor" said "when a patient presents himself to me to have his teeth filled, whose mouth is stinking and filthy, I never touch it, but send him away, until his mouth is restored to a healthy condition." Any one would mentally ask, "how is the man's mouth to be rendered healthy if the Dentist never touches it?" Perhaps we should charitably believe that such writings are the result of carelessness only; but they are none the less confounding on that account.

It is only required that statements be intelligently written down, and that nothing be written but facts, when it is the intention to instruct, or communicate results. Let that which has been done be stated as it was done, in language that no one can misapprehend, or fail to understand. I have read many statements of practice that I could not for my life follow out. For instance, I have never filled the fangs of a molar tooth "quite to the apex;" and I take this occasion to say that I don't believe any other man ever did with metal. And then when a Dentist says over his signature that he injected fluid into the nerve cavity of a necrosed tooth, which was passed out through the gum opposite the point of the fang, and that he continued his treatment in this way until "all the parts were restored to a healthy condition," and then filled the tooth fang and all, and that it continued to do well. I am satisfied of the fallacy of the whole statement. I don't like such loose language. If the writer had said he filled the tooth, including the fang, and that the fistulous opening that gave exit to his injected fluid remaining open, discharging puss through the gum, there was no active inflammation, and therefore the tooth "continued to do well," he would have stated the case correctly and intelligently. It can not be possible that he wished anybody to believe that the denuded alveolus and point of the fang were reclothed with periosteum, healthy periosteum, transmitting life and nutrition to the restored bone, and otherwise performing all the functions of organic animal life, which must have been the case, if the parts were all "restored to a healthy condition." And further, I must be allowed to say, that I am satisfied that more has been written and said about fang filling than the practice will justify. But, I do not propose to raise questions, and will pass this matter, allowing every one to do what he can in that difficult business. I will say, however, *en passent*, that until we can learn to restore the wanted vitality to nerveless teeth, and become skillful enough to obliterate all space, "quite to the apex of the fangs," we must be prepared to meet with failures in fang filling, as a means of keeping the teeth validly in the mouth.

To me, there is nothing reasonable in the idea that the oxychloride of zinc can induce the formation of natural bone over exposed pulps. It is a foreign substance, and an irritant; and I have found in several cases where I used it under favorable circumstances, that at the end of a few months, or a year afterwards, the pulp was all gone, and there remained simply a necrosed tooth. A few cases where I have applied it to newly exposed nerves, have resulted in toothache, more or less violent, which, when not seen in time, ended in death of the nerve, periostitis, swelled face, and sometimes in abscess. But some of my cases are going on well yet. Still, the oxychloride promises to become a useful agent in our hands; and I love to use it under my fillings when I know that they approximate the membrane. I use it thus because of its non-conducting qualities. In sensitive dentine it has also proved of great value to me. I have applied it in cavities where it was impossible to cut away, and in a few hours, days or weeks, as circumstances favored, have performed the operation of excavating with no pain whatever.

My success in attempting to save nerves alive, after exposure from any cause, has not been satisfactory; nor do I believe so desired a consummation is likely to be reached, except in a very limited number of cases, unless our knowledge of vital chemistry should be greatly enhanced. The nerve of a tooth is a tissue altogether too delicate and sensitive to sustain quietly the near neighborhood of inorganic matter, or rapid conducting bodies. I regard all such cases as perplexing, and would rather not see them come to my office; and I feel that until some new and more successful method of treating them comes up, it is safest to destroy the pulp at once, and take the chances of fang filling under the best facilities that can be devised. And here, lest I should be misunderstood upon my previous remarks, I say that I approve of fang filling, as far as it is practicable; though experience has taught me that great pains taking is required in its conduct. I first pack with cotton and creosote as far as it can be pushed, and as firmly as possible, and let the case remain for an indefinite period, or until I feel that no danger is iminent, when I complete the filling with tin foil. If all is well at the end of a few months, I remove the tin and substitute gold, and seldom have trouble afterwards, if all my manipulations have been carefully conducted. My reasons for using tin are, that in the event of threatened mischief from pent up fluid, or gas, it is more easily removed. It is, therefore, a trial filling. And I never let a patient go away with the understanding that teeth thus conditioned are not in perfect harmony with the general system, and that periostitis may set in at any time, when the equilibrium of the vital forces becomes broken.

I abhor misunderstandings. We have trouble enough in combating ignorance, without ourselves bringing more, either by stating too much or too little. We must deal fairly but firmly with our patients, at the same time that we avoid that egotistical air which says, all " over us " I know it all, what do you know?" And then, it is just as easy to err on the other extreme by attempting to talk a patient into a new set of teeth, or the necessity for a certain amount or kind of filling, or other operations, by mere volubility of words or strategy. Both these courses offend all persons of good sense, and seldom fail to inspire the idea that the Dentist has more intention towards the purse than the real good of the mouths of his patients. There is a class that can be talked into any measure, I know; but reaction always comes at last, and these very persons will, when sober reflection takes possession, abuse or praise the Dentist in the proportion that his promises have been realized or otherwise. Everybody finds out if they have been deceived, and if they see that it has been done for money, nothing on the part of the deceiver will ever again secure their confidence.

The whole world dislikes and shuns mere mercenary men; for every individual wants disinterested friendship, and will pay cheerfully its equivalent, either in money or like good deeds. If there are, therefore, men who have entered the Dental profession under the dominant idea of getting money quick and easy, and with little capital, they are in the wrong place, and so far as found out, ought to be shunned and neglected. He is a poor judge of human nature, and even of his own interests who has deemed it necessary to play upon the credulity of people in order to secure patronage and money and reputation. Honest merit is always compensated, while those advantages that the demagogue calls about him are as the morning dew. And why should I envy my neighbor because he is my competitor in business? He may be my superior in some things, and I may gain something by conferring with him, and he may learn of me; and if the people see that we are trying to sustain each other, they will think the more of us both. And under such circumstances the public patronage will be distributed fairly. No man can secure all the business of his community in anything; the world is not arranged on that basis. Pure intentions and honest conduct will always be rewarded; and I shall hold that if a Dentist is as honest as he ought to be, he will not desire business when he is conscious of his incompetency, but turn his attention to some pursuit for which his talents fit him.

There is no consolation of life so sweet as that which springs from perfect self approval; or in other words from being satisfied with one's self. Our unintentional failures are goading and depressing, whether they be of a moral character, or belonging to the business and temporal affairs of life; but how much more so those that have been done with a will, when the unperverted moral sense comes to see them. Did you ever feel a sweeter complacency than that which follows a day well spent; a day in retrospecting which no act of yours rises up to condemn; a day in which you have been perfect in all your manipulations about the mouth; and have discharged every moral, civil and social obligation to all you have been called into commerce with; and have received the pecuniary remuneration due to your services?

But these are pictures I don't like to dwell upon; and I would not parade them before the reader, but for the hope that some may see their image reflected, and be all the better for the sight.

It has chanced to me, as well as others, to fall in company with Dentists who, during an interview more or less extended, could find no theme of discourse, or object of thought outside of or above themselves. They are not long in letting you know what their monthly business will foot up, which is sure to be a pretty large figure, if you take the verbal statement in place of the day book, which is rarely produced. And then they have done over so many plate jobs that somebody else had made, and which could not be worn, besides refilling most of the teeth that had been filled round about, and always charged high prices too, which of course is to be taken as evidence of merit and skill; and they have patients coming from long distances; and such is the rush of patrons that they never get to dinner, and have hardly time to be civil to friends; and if no one happens to drop in while you are there, "it is the first time it has happened so for six months." And they have ways and methods of doing things that are all their own; and somehow, when the interview ends, you are just as wise as when you came, so for as your friends ways and methods are concerned. You may, if you choose, be overawed by the superiority of your brother professor; and you may likewise feel a little dampened if you have tried to talk some too, to find that after you have seized an interval to force in a few sentences, your companion remains utterly oblivious of what you have said, and chimes in again at the very point where you suspended him. One man who had really attained to some celebrity as a Dentist, had his workshop in the garret, which was a sealed department to all besides himself.

And again, the brains of some self sharpener has worked out an invention that is to be of real or imaginary benefit to the facilities of the Dental art, and with the speed of the locomotive it travels to Washington to secure letters patent, that all its ghostly or material pecuniary issues may be alone to the inventor. There may be a difference of opinion on this practice of patenting Dental inventions. One thing at least must be conceded: discoverers and inventors are a useful class of men; but if anything useful is found out in Dental appliances or material, and the secret or right to use is to be thrown into the market, under the protection of law, to enrich the discoverer, it is simply a mercenary view of the subject. I sincerely hope that our Associations will set their seals of disapproval on all Dental patents; and if the seal includes the patentee all the better.

The question, "how much money can I make out of my profession?" is a low conception of life and its responsibilities. But it is to be feared that, impelled by the frantic rage to get wealthy which marks the times, many may be tempted to depart from honorable professional life, in order to make the most of their cases. In this way I have sufficient reasons for believing that millions of teeth are annually pulled ruthlessly from their sockets in order that the Dentist may supply their places with artificial sets, with a moiety of the pains taking, labor and time that it would have required to treat and fill the natural ones, and at the same time gain larger profits.

This practice I hold to be just a little less than criminal, in cases where the teeth could have been patched and mended, and made to answer the purposes of mastication. It is our first and highest duty to save and not destroy. I am aware, however, that we all have to extract many teeth in the course of practice that could be saved in some condition of use and harmlessness in the mouth, for a greater or less space of time. This happens in the case of persons who come to us with tooth-ache, and must have relief, and who we know own neither the means nor inclination to have anything done beyond getting rid of a painful tooth. In such cases the extraction of the teeth without regard to what might be done to retain them is right.

The professional Dentist should be a gentleman in the fullest meaning of the title. He should be a Christian gentleman. His work is plain before him. He does not have to feel his way. That which he has to do can be done, and he can comprehend it all. The Dentist is to make himself personally agreeable; ever pleasant to his patients; in manners as well as in the delicacy and gentleness of his manipulations of the teeth. When the infliction of pain will be unavoidable, kind and aspiring words will take away much of the dread, besides inspiring necessary confidence in the operator; while stern, brusque and unfeeling deportment will not fail to produce the very opposite state of things.

It has always been my rule not to promise too much, and in the long run I have approved my course in this respect. An excellent lady, for whom I had filled many teeth called to have the left inferior *dens sapientia* extracted. It was a shell, and at the time, suffering from acute periostitis. I told her that the extraction would be painful without anæsthesia, and that if the fangs were curved, there was a possibility that the crown might break. She was afraid of me as a tooth extractor, and went away. On the following morning she returned, with the crown of the tooth broken away and after having suffered a night of excruciating pain. She regretted, she said, that she had not rested in my opinion. I think it right to deliver a candid opinion in all cases. It will be best for our patients, and ourselves in the long run.

And finally, it would greatly promote the character and pecuniary remuneration of Dental practice, if there were a fraternal co-operation of all Dentists. On the other hand, professional bickerings and jealousies are, and will be a dead weight, lowering the reputation of Dentistry; because the people have the right to doubt the competency of men who are trying to build themselves up by traducing others. The idea can not be otherwise, under such circumstances, than that money is the dominant principle.

